# Genome-Wide Association Analysis for Hybrid Breeding in Wheat

**DOI:** 10.3390/ijms232315321

**Published:** 2022-12-05

**Authors:** Monika Mokrzycka, Stefan Stojałowski, Mirosław Tyrka, Przemysław Matysik, Barbara Żmijewska, Rafał Marcinkowski, Urszula Woźna-Pawlak, Róża Martofel, Michał Rokicki, Monika Rakoczy-Trojanowska, Paweł Krajewski

**Affiliations:** 1 Institute of Plant Genetics, Polish Academy of Science, Strzeszyńska 34, 60-479 Poznań, Poland; 2 Department of Plant Genetics, Breeding and Biotechnology, West Pomeranian University of Technology Szczecin, Słowackiego 17, 71-434 Szczecin, Poland; 3 Department of Biotechnology and Bioinformatics, Faculty of Chemistry, Rzeszow University of Technology, Powstańców Warszawy 6, 35-959 Rzeszów, Poland; 4 Plant Breeding Strzelce Group IHAR Ltd., Główna 20, 99-307 Strzelce, Poland; 5 Poznań Plant Breeding Ltd., Kasztanowa 5, 63-004 Tulce, Poland; 6 Plant Breeding and Acclimatization Institute, Radzików, 05-870 Błonie, Poland; 7 Department of Plant Genetics, Breeding and Biotechnology, Institute of Biology, Warsaw University of Life Sciences, Nowoursynowska 159, 02-776 Warszawa, Poland

**Keywords:** genome-wide association study, wheat hybrid breeding

## Abstract

Disclosure of markers that are significantly associated with plant traits can help develop new varieties with desirable properties. This study determined the genome-wide associations based on DArTseq markers for six agronomic traits assessed in eight environments for wheat. Moreover, the association study for heterosis and analysis of the effects of markers grouped by linkage disequilibrium were performed based on mean values over all experiments. All results were validated using data from post-registration trials. GWAS revealed 1273 single nucleotide polymorphisms with biologically significant effects. Most polymorphisms were predicted to be modifiers of protein translation, with only two having a more pronounced effect. Markers significantly associated with the considered set of features were clustered within chromosomes based on linkage disequilibrium in 327 LD blocks. A GWAS for heterosis revealed 1261 markers with significant effects.

## 1. Introduction

Wheat (*Triticum aestivum* L.) is an important source of calories and proteins for humans worldwide [1]. This crop is cultivated on approximately 216 million hectares and yields over 765 million tons of grain [2]. For the last 60 years, the average yield of wheat has been increased 3-fold to the current level of approx. 3.5 t/ha [2,3], but the progress in this regard has been relatively slow in recent years. Norman Borlaug’s “Green Revolution” initiated this continuous progress, which aimed to release new highly productive cultivars for developing countries. The use of heterosis and hybrid wheat breeding is a possible option for maintaining or boosting yield productivity in modern wheat cultivars. The possibility of the commercial use of wheat F1 hybrids was suggested as early as 1963 by Briggle [4]. Wheat hybrids may significantly outperform traditional cultivars regarding yield level (usually by under 5%, but in particular situations by more than 40%; [5]) because of the effect of heterosis and can reveal higher stability in various environments due to their heterozygotic nature [6,7,8]. Moreover, the certified hybrid seeds desired by producers protect the interests of breeders [9].

The availability of high-throughput genotyping techniques, such as genotyping by sequencing (GBS) or single nucleotide polymorphism (SNP) arrays, has accelerated global genetic analyses of large genome species, such as wheat [10,11,12,13,14]. The results of genome-wide association studies (GWAS) can be applied to the selection of the most valuable genotypes (genomic selection). GWAS was recently used to analyze anther extrusion in wheat [15,16]. Association studies based on various types of populations for the wheat plant traits analyzed in this report were also performed; we refer to a number of them in Discussion, Section 3.1, Section 3.2 and Section 3.3. More complex analyses focusing on the multi-trait requirements of hybrid breeding have not yet been reported. In the breeding of traditional cultivars, the target of GWAS is relatively clear, and the objective of genomic selection is to identify genotypes with alleles associated with increased values of yield-related traits that are frequently affected by environmental changes [17,18,19,20]. The application of GWAS for genomic selection in hybrid breeding procedures is complex. The successful release of a cultivar results from selecting a proper maternal line (seed parent), paternal line (pollen parent), and prediction of the heterosis effect revealed by the final hybrid. The challenge is that a desirable trait in one of these three components can be undesirable in another. Furthermore, the best performance of the final hybrid should be achieved when numerous loci of high-yielding parents remain heterozygotic. Genomic selection in hybrid breeding should be adjusted to meet these requirements.

In addition to the agronomic performance of related traits, the condition of heterosis breeding success is the appropriate flowering biology of the parental components. Different elements of flowering biology determine the suitability of male and female wheat parents for hybrid breeding. Efficient anther extrusion and a high quantity of released pollen over an extended period are considered the most important traits desired in male candidates for the successful production of hybrid wheat seeds. For female parents as pollen recipients, more significant is the ability to open flowers in the time necessary to get pollinated for extended periods, coupled with hairy stigmas. Considering the plant height, it is recommended that the pollen parent be taller than the seed parent because the pollen of wheat is much less mobile than the pollen of typical wind-pollinated plants such as maize and rye [3,7,16,21,22,23].

Despite several studies, the processes of flower opening and anther extrusion, a very complex and dynamic phenomenon combining anatomical, physiological, and biochemical parameters, are still not fully understood or defined [24,25,26]. Nevertheless, the variation and heritability of flowering-related traits are considered moderate to high, which provides the opportunity to select desirable parental components [7,27].

In the presented work, we use the data presented in [28], which was applied to the analysis of the genetic structure of a large pool of 509 wheat varieties and breeding lines. On adding to them the phenotypic observations obtained in two independent series of field trials, we presume that by using GWAS we will identify chromosomal regions associated with six agronomically important traits that are significant for wheat hybrid breeding.

To verify this presumption, we set the following objectives: (1) to apply the genotyping data [28] consisting of SNP marker observations determined by the DArTseq platform for GWAS, and (2) to identify chromosomal regions associated with six traits within a set of wheat genotypes across different environments.

## 2. Results

### 2.1. Phenotypic Characteristics of the Accessions

Six phenotypic traits were assessed in eight environments. The distributions of genotypic means (BLUPs obtained in individual experiments; Table S1) were approximately normal; a slight skewness to the right was observed for time of flowering—FT (Figure S1). Two phenological traits, time of heading—HT and FT, were positively correlated with each other and with plant height—PH (*p*-value < 0.05; Figure S1). In contrast, for PH, a negative correlation was observed with yield-related traits (number of kernels per spike—KN, weight of kernels per spike—KW, and thousand kernel weight—TKW). KW was positively correlated with TKW.

In ANOVA, all fixed effects, i.e., effects of the year (Y), localization (L), and Y × L interaction, were significant (at least at a *p*-value < 0.05, but most of them at *p*-value < 0.001). The variance components for random effects of genotypes (G) were much larger than those for G × Y and G × L interactions for traits HT, FT, and PH, which was reflected by the large heritability for these traits (88–90%; Table 1). The variance components were in the same order for yield-related traits, and therefore, heritability was smaller (38–54%).

**Figure 1:**
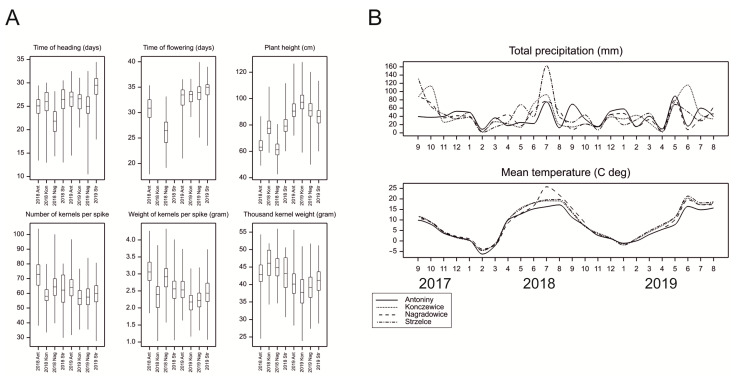
(**A**) Distributions of genotypic means in experiments; Ant—Antoniny, Kon—Konczewice, Nag—Nagradowice, Str—Strzelce; (**B**) Observations of weather parameters in 2017–2019 at four locations of experiments (ten-day summaries).

A comparison of the distributions of genotypic means in particular experiments showed that accessions headed and flowered earlier and were shorter in 2018 than in 2019 (Figure 1A), and that the yield parameters were higher in 2018. The weather conditions, as characterized by monthly precipitation and temperatures, differed slightly between the two growing seasons. In 2017/2018, winter temperatures were lower, and summer precipitation was slightly higher than in 2018/2019 (Figure 1B).

Stability analysis in the AMMI model revealed that, in general, the reactions of genotypes to conditions in the four locations were more similar in 2019 than in 2018 (Figure S2A). In 2018, for traits HT, PH, KN, and TKW, a considerable difference existed between reactions to conditions in (Antoniny, Nagradowice) vs. (Konczewice, Strzelce). The patterns of the interactions for localization were similar for HT and TKW. Genotypes showed continuous distributions of interactions. For FT accessions, PHR_6 (Akteur), STH_148 (UKR 12), and for KW accessions, STH_65 (STH 9025), STH_167 (Cornea ost.) showed outlying instability. The largest significant correlations existed between instability variances for genotypes estimated for pairs of traits: HT and FT and KW and TKW (Figure S2B,C); correlations for trait pairs (PH, FT) and (KW, KN) were statistically significant but very small. The ranks of genotypes concerning instability variances for all traits are presented in Table S2; they show that the most stable genotypes for one trait were usually much less stable for the other.

### 2.2. Association Analysis

GWAS revealed 7603 SNPs with polymorphisms significantly associated with at least one phenotypic trait (only SNPs with more than five accessions in each of the two homozygous classes; BH corrected *p*-value < 0.05). The characteristics of the allelic substitution effects for these loci are shown in Table 2.

**Figure 2:**
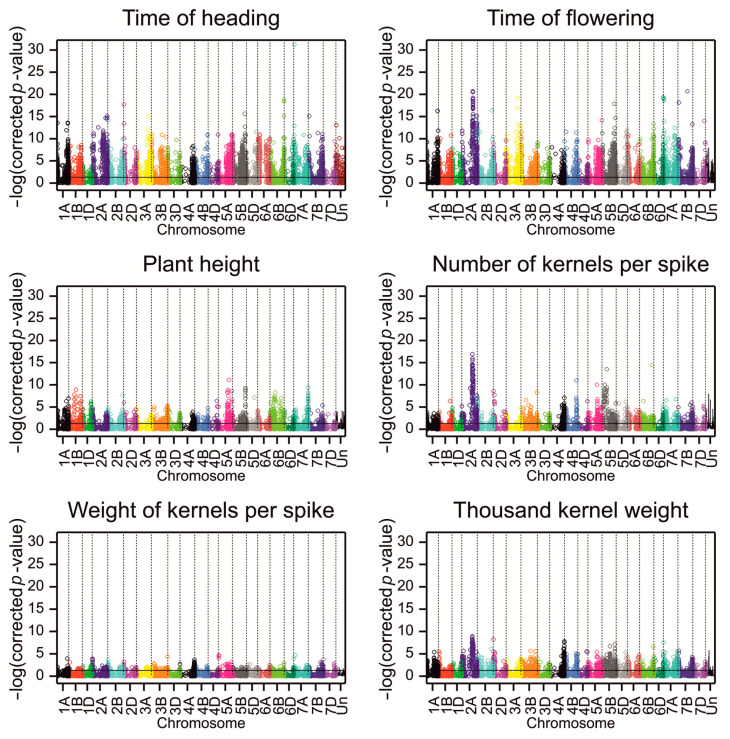
Manhattan plots for associations of SNPs with studied phenotypic traits. Points represent *p*-values for associations corrected by the Benjamini–Hochberg method. The black line visualizes the critical significance level of 0.05.

By considering also the criterion related to the size of the substitution effect being in the second lower or upper percentile, the number of associated markers was reduced to 1273, and the number of significant associations was reduced to 1344 (Figure 2, Table 3 and Table S3).

**Figure 3:**
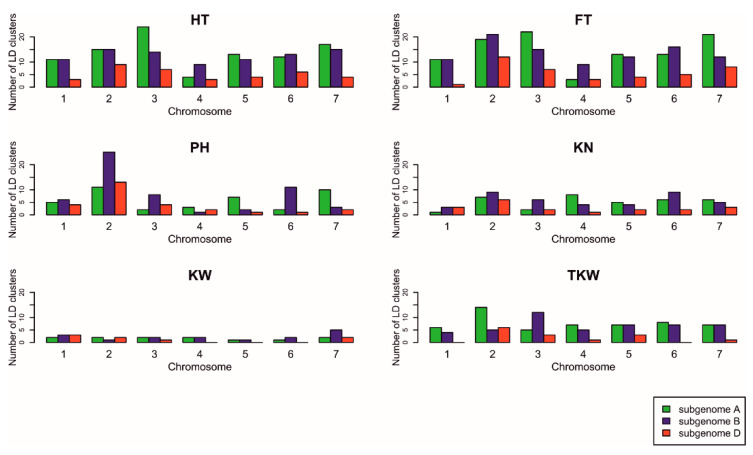
Number of LD clusters containing associated SNPs by chromosomes for each trait.

The mean linkage disequilibrium (LD) with other SNPs in the region ±5 Mb was determined for significant SNPs. A detailed analysis of LD has been provided in [28] (a heatmap of LD on chromosome 1A in Figure S2A, exemplary LD clusters in Figure S2B, and the plots of LD vs. physical distance between markers with 0–20 Mb distance intervals in Figure 5A of [28]). The percentage of associations with a small mean LD (<0.01) in this interval varied from 5% for PH to 20% for KW (Table 3). The significant SNPs were clustered into small groups of no more than five markers within chromosomes based on LD (Table 3). The highest number of LD clusters was on chromosome 3A for the two phenological traits, on chromosome 2B for PH, on 2B and 6B for KN, on 7B for KW, and on 2A for TKW (Figure 3).

The fraction of associations showing interaction with the environment was the lowest for PH and KN and the highest for KW. Associations were unevenly distributed in the wheat subgenomes. Most of the associated SNPs mapped to the A and B subgenomes (38.4% and 45.01%, respectively), whereas only 14.14% were derived from D, and 2.44% remained unmapped (Table 3, Figure 2); however, the proportion of the relative number of SNPs was similar: 8.99% from the A subgenome, 10.14% from B, 8.45% from D and 11.07 from Un. For five traits, most associations were detected in subgenome A, and only for PH were most associations detected in subgenome B. More than 50% of the associated SNPs for all the traits were located in genes. Most SNPs associated with traits were predicted to modify protein translation, and only two SNPs significantly influenced the protein structure. The first SNP (1130302|F|0-44|TG), which was associated with FT, altered the splicing donor site in TraesCS6A02G085100 (Arabidopsis RPT3 ortholog), which is responsible for ATP-dependent degradation of ubiquitinated proteins. The second SNP (1090593|F|0-44|CT) was associated with TKW and introduced a STOP codon in the gene TraesCS4B02G086500, annotated with “carbohydrate metabolic process” and “polygalacturonase activity” GO terms.

Allelic substitution effects for different traits were correlated (Figure S3). However, not all correlations were of the same size and sign as those of the traits themselves. For example, allelic effects for PH were negatively correlated with effects for phenological traits (positive correlation for traits), and effects for KW were positively correlated with phenological traits (negative correlation for traits).

**Figure 4:**
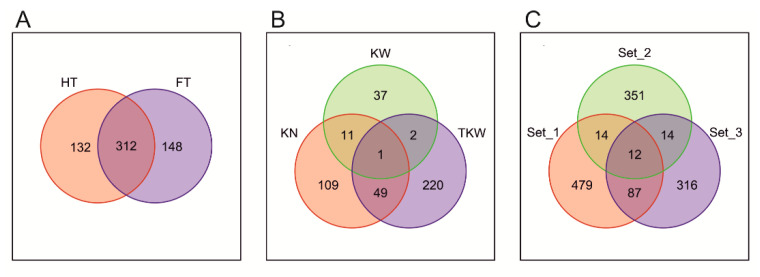
The number of common and specific SNPs in (**A**,**B**) association sets for traits and (**C**) association set 1 (HT or FT), set 2 (PH) and set 3 (KN, KW or TKW).

SNPs associated with traits were divided into three sets corresponding to plant phenology (HT or FT, set 1), PH (set 2), and kernel properties (KN or KW or TKW, set 3), with the numbers of associations in these sets being 592, 391, and 429, respectively. There were 26, 99, and 26 SNPs common to sets 1 and 2, 1 and 3, and 2 and 3, respectively, and the 12 SNPs common for all three sets (Figure 4, Table S3). For pairs of traits, the largest number of common significant SNPs was recorded for HT and FT (312), which is consistent with a large correlation between these two traits reported above.

Of the 26 markers that belonged to sets 1 and 2, 17 were assigned to genes, with 5 being modifiers and 4 substitutions having moderate effects (Table 4). There were three markers with concordant effects on earliness and plant height, with the modifying effect of substitutions. Two of these (2253029|F|0-10|CT and 1237800|F|0-13|CG) were associated with genes (Table S3). Out of 12 SNPs common for sets 1, 2 and 3, eleven were associated with KN and one with TKW. The same direction of effect could be seen for FT and KW, while the opposite direction was seen for KN and PH.

### 2.3. Marker LD Clusters

**Figure 5:**
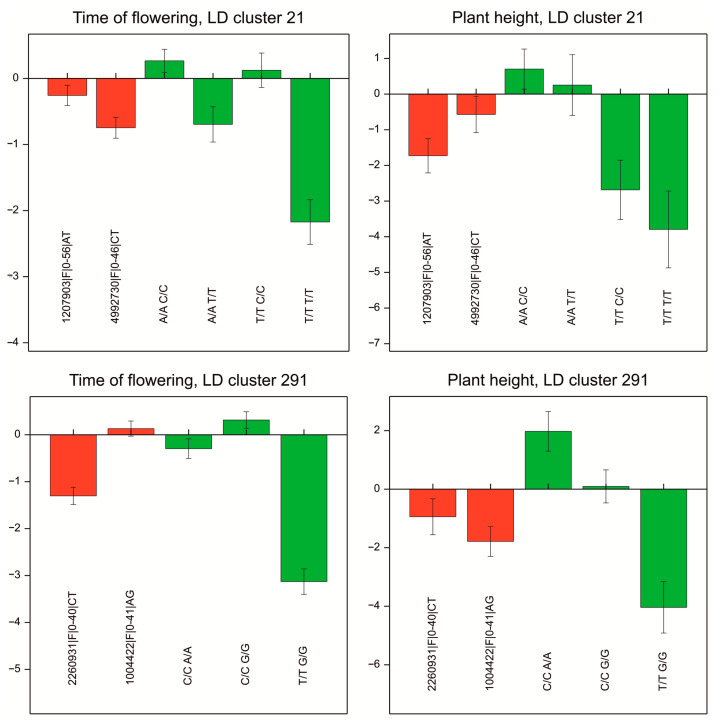
Effects found in the association analysis: red—allelic effects of individual SNPs, averaged over eight experiments; green—haplotype effects (deviations from the general mean). Bars represent standard errors of effects. Illustrated are the results for two marker clusters with effects concordant for earliness (FT) and plant height (PH).

Clusters of markers significantly associated with HT, FT (set 1), and with PH (set 2) (Table 3) were considered for LD-based GWAS. Combinations of their genotypes, represented by at least 25 accessions from the investigated collection, were considered for further analysis. The characteristics of the 327 marker clusters are presented in Table S4. Among them, we attempted to identify the effects of genotypic combinations that were higher than the separate allele effects of particular single markers. Examples of genotypic combinations with large negative effects on FT, HT, and PH are shown in Figure 5. For marker LD block no. 21, which clusters markers “1207903|F|0-56|AT_4992730|F|0-46|CT”, the variant “T/T T/T” was present in 26 accessions and was related to early flowering (effect of −2.9 days) and low plants (effect of −3.8 cm) in comparison to the average of all accessions. One of these markers was assigned as an upstream modifier of the gene TraesCS1B02G028100, annotated as involved in “serine-type endopeptidase inhibitor activity.” Other interesting marker clusters with higher effects contributed by haplotypes were identified, for example, no. “291”, 2260931|F|0-40|CT_1004422|F|0-41|AG. We noted that 76% of the genotypic variants in marker LD blocks were fully homozygous. Therefore, their information was equivalent to the knowledge of haplotypes.

### 2.4. Heterosis

**Figure 6:**
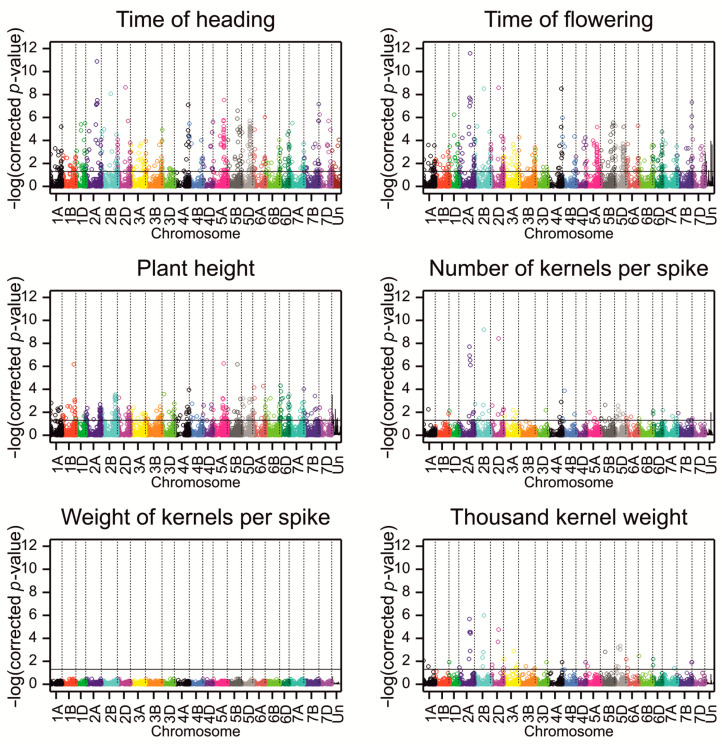
Manhattan plots for heterosis effects of SNPs. Points represent *p*-values for associations corrected by the Benjamini–Hochberg method. The black line visualizes the critical significance level of 0.05.

Searching for heterosis of SNP polymorphisms (on the set of SNPs with more than five heterozygous lines; BH corrected *p*-value < 0.05) revealed 1261 SNPs with significant effects (Table 5 and Table S5), with the largest number of effects for phenological traits and no effects for KW. Significant SNPs were evenly distributed over the subgenomes, with 53–80% of SNPs in the genes (Figure 6). None of the SNPs revealed a high translation effect, and the majority of the predicted effects were classified as low or modifying protein translation.

Association sets for traits (HT, FT), (PH), (KN, KW, and TKW) based on heterosis effects contained 480, 324, and 83 markers, respectively. The latter (yield-related) set contained 52 markers assigned to genes; the heterosis effects in this set were: from −4.50 to 3.09 grains for KN, from −0.12 to 0.09 g for KW, and from −2.09 to 4.13 g for TKW. The highest negative effect for KN (−4.50 grains) was for marker 3024735|F|0-10|GA (TraesCS7B02G481400).

The correlations of heterosis effects were similar to those of additive effects (Figure S4).

### 2.5. Allelic Substitution Effects vs. Heterosis Effects

**Figure 7:**
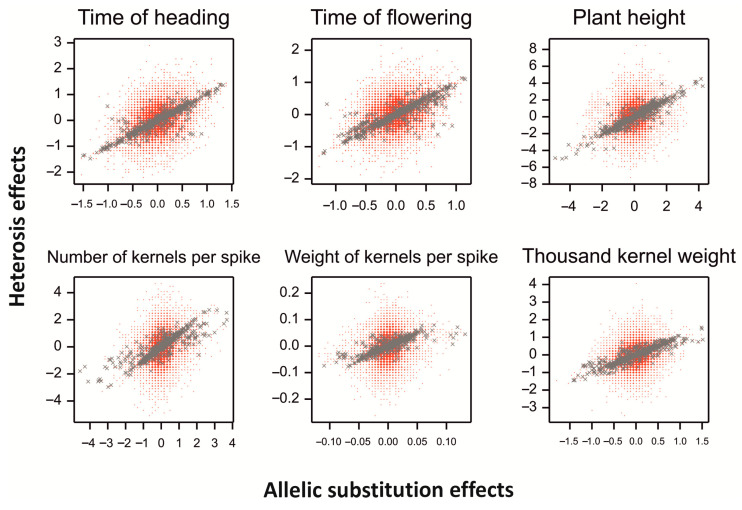
Heterosis effects vs. additive effects for six phenotypic traits; gray points for markers with heterozygosity > 0.5.

Allelic effects were correlated with heterosis effects for markers with high heterozygosity (Figure 7). The range of additive effects was smaller than that of heterosis effects for all traits.

One marker, 1238701|F|0-16|GA (TraesCS2D02G127300), belonged to sets 1 and 2 with respect to additive effects (effects for HT −0.75, for PH 2.47), and to set 3 with respect to heterosis effects (effect 2.25 for TKW).

### 2.6. GO Annotation of Associated Markers

GO terms (biological processes) represented in sets of genes assigned to SNPs in association sets 1, 2, and 3 for allelic effects and heterosis effects are shown in Table S6A and Table S6B, respectively. Most frequently (16–26%), markers associated with both additive and heterosis effects were involved in redox processes and protein phosphorylation. The next most common GO terms were regulation of transcription (DNA-templated), carbohydrate metabolic process, and transmembrane transport. Responses to auxins were identified in groups of genes responsible for plant height additive effects more frequently than proteolysis.

### 2.7. Validation of Results Using Post-Registration Trial Data

**Figure 8:**
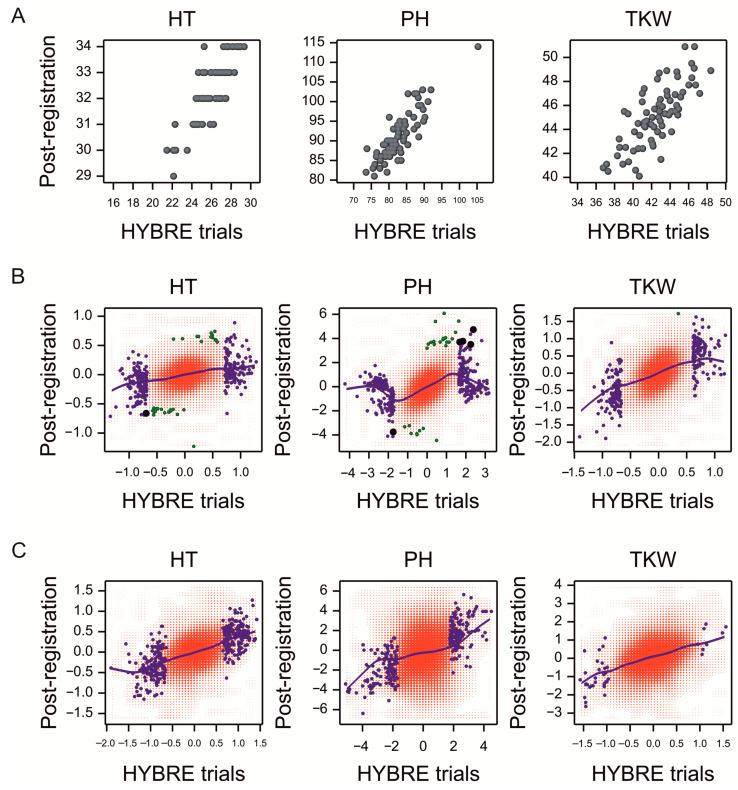
(**A**) Scatterplots of varietal means observed in post-registration experiments (PRT) vs. means observed in HYBRE trials, for 75 varieties occurring in both series of experiments. (**B**) Density plots of allelic effects for markers estimated in post-registration experiments (PRT) vs. effects estimated in HYBRE trials in the set of 75 varieties. (**C**) Density plots of heterosis effects estimated in PRT vs. HYBRE. (**B**,**C**) Blue—significant in HYBRE, green—significant in PRT, and black—significant in both analyses. The blue lines are trend lines determined by smooth regression using the thin plate method.

Data from post-registration trials (PRT) were used to validate the GWAS results obtained in the HYBRE experiments. The general varietal means of HT, PH, and TKW from the two series of experiments were correlated (0.84, 0.86, and 0.76, respectively; *p*-value < 0.001; Figure 8A). Allelic effects from GWAS were also correlated (0.29, 0.42, and 0.40, respectively; *p*-value < 0.001; Figure 8B). However, only three SNPs were significantly associated with traits in both series of trials: one for HT (3024420|F|0-9|CT, modifier, intergenic), and two for PH (1087592|F|0-37|GA, modifier, downstream of TraesCS5A02G277900 and 1126438|F|0-22|CT, modifier, intergenic). The heterosis effects were also correlated (Figure 8C).

## 3. Discussion

The main goal of hybrid wheat breeding is to exploit the heterosis effect. Final success depends on several factors, but yield is a crucial goal. Without a significant increase in productivity offered by hybrid cultivars, they will not be the choice of farmers; as an alternative, they may apply less expensive seeds of well-performing classic cultivars. The cost of hybrid seed production is also important. It depends on numerous factors related to plant morphology and the biology of flowering [3,7,30]. The cross-pollination of wheat plants is based on the wind, but it is significantly less effective than that of typical open-pollinated plants, such as rye and maize. Waines and Hedge [31] noticed that singular wheat pollen could be found as far as 1000 m from the plantation, but hybrid seeds are usually undetectable beyond 20–30 m. The distance between parental lines of wheat allowing for economically sufficient effectiveness of seed production is limited to 2–3 m, and larger values can be applied when the pollen parent is taller than the seed parent. The reduction of plant height in wheat (highly recommended for maternal components) can be achieved by utilizing properly selected reduced height (Rht) genes.

In this study, we present a multifaceted statistical analysis aimed at providing information on a broad population of cultivars and breeding strains, which can primarily be used in wheat hybrid breeding. We investigated other morphological and phenological traits significant for efficient seed production and chosen traits related to wheat productivity, aiming to verify whether our non-standard genome-wide association study methods have potential application in hybrid breeding of wheat.

The analysis of variance showed that genotypic variability, measured in relation to variabilities caused by genotype by year and genotype by localization interactions, and, consequently, broad-sense heritability, was higher for phenological traits and for plant height than for yield-related traits. This indicates the possibility of selecting valuable parental forms independently, to some extent, from the target environment. Additionally, stability analysis (AMMI) showed that, although a given genotype may be subjected to various degrees of environmental variance for various traits, some correlation exists among instability variances for traits related to hybrid component selection.

However, our association analysis was performed assuming that SNP variant effects can also interact with environmental conditions. This aspect of GWAS analysis is often addressed by performing separate analyses for each environment (cf. [32,33,34]), mainly because of the limited options presented by the data analysis software. Based on the mixed model developed by Malosetti et al. [35], our approach allowed for the explicit testing of markers by environment interaction effects, which provided a conclusion on the lower environmental variability of SNP effects for plant height than for phenology. We also performed GWAS analysis using marker LD clusters. The most promising marker clusters were those for which the effects of genotypic combinations were higher than the allelic effects of individual markers. Furthermore, concordant effects of earliness and plant height are required for the practical importance of the haplotype. The applied procedure allowed the identification of groups of markers with a high impact on phenotypic traits, which could be expected for individual polymorphisms. Thus, we demonstrated the possibility of using the phenomenon of non-allelic interactions in LD-informed hybrid breeding. Moreover, as hybrid breeding is based on the effects of heterozygous materials, another association analysis was performed with respect to heterosis. It aimed to identify genomic loci for which heterozygotes could be more profitable than homozygotes, especially for yield-related traits. Although the results obtained in this way are far less valuable than those that could be gained from observations of hybrids, this is undoubtedly progress compared to the methodological approaches used to date in this type of research.

Validation of GWAS results can be done by an independent experimental study concerning another pool of genotypes. In our case, the pool covers most of the accessions that are practically interesting for project stakeholders. Another form of validation can be based on a real breeding process; this has been started by passing our results to breeders, who have already performed selected crosses and are currently assessing the value of hybrids by their standard procedures. In this report, to verify the results of GWAS, publicly available data from independent, post-registration trials performed in a wider set of environments were used. The mean BLUPs for genotypes from the HYBRE experiments were correlated with the general means from the PRT. Moreover, substantial correlations between allelic substitution effects obtained from GWAS on both datasets were obtained; the same was true for heterosis effects. The effects concerning plant height were found to be the most correlated. This means that the data obtained in the HYBRE experiment are representative, and it can be assumed that new breeding creations can be evaluated in limited field experiments rather than in large experimental systems.

### 3.1. Flowering-Related QTLs and Genes

The expected female component of the hybrid should contain long stigmatic hairs that are fully extruded and receptive for long periods [7]. Wheat stigma remains receptive for up to 13 days after anthesis; however, it is usually highly receptive for no more than 3 days after anthesis [21]. Effective anther extrusion of the pollen parent is crucial for the successful setting of hybrid seeds. Recently, Denisow et al. [36] showed that the anther extrusion plays a much more important role in the contribution to the final amount of pollen available for cross-pollination than previously thought. Flowering generally begins at the center of a wheat spike and proceeds in both directions (up and down). Within each spikelet, the primary floret opens first, followed by the secondary, tertiary, etc. The first two florets of the spikelet produce the most abundant anthers, filled with the most viable pollen [37]. Thus, it is suggested that when these highly effective florets of the male component begin flowering, the female parent should be ready to receive pollen. The optimal difference between the flowering times of the seed parent and pollen parent is 2–3 days [30]. The phenotypic efficiency of some of the SNP markers indicated in our analyses met these criteria. The studied set of wheat accessions had been focused on genotypes cultivated in Europe [28]. Therefore, phenotypic variation was, in some way, limited. Despite this, some of the selected SNP markers revealed additive effects ranging from 1–1.6, corresponding to a difference from 2 to over 3 days of flowering time of homozygotic wheat genotypes, which are potential parental components of hybrids.

Markers associated with flowering time are necessary to synchronize the flowering of hybrid wheat components. Eight QTLs for heading time have been reported [38]. One of them, QHD_7A_psr_ParW670_CFLN17, corresponding to a relatively short region 710–719 Mbp on 7A, colocalized with the 3025631|F|0-10|GA marker found in our study. The regulation of flowering has been extensively studied at the gene level. Flowering is mainly regulated by vernalization (VRN1, VRN2, VRN3, and VRN-D4), photoperiod (Ppd-A1, Ppd-B1 Ppd, and Ppd-D1), and earliness per se (eps) genes [39]. Some of these genes showed pleiotropic effects, i.e., Ppd-A1 increased TKW and yield. Similarly, Ppd-B1 was associated with a high kernel number [40]. Eps genes interact with Ppd1 and are associated with spikelet number [41]. In wheat, a homolog of the Arabidopsis early-flowering 3 (ELF3) gene was identified as a candidate gene for Eps-Am1 [42]. Additional genes affecting flowering time in wheat were reviewed by Zhang et al. [39].

Searching Ensembl Plants for wheat orthologs of 204 Arabidopsis thaliana genes related to flowering [43] resulted in a list of 617 wheat genes. Of these, 31 were assigned to some of the SNPs analyzed in this study. Five SNPs were associated with FT or HT, with a negative effect of the ALT allele; these were orthologs of the A. thaliana genes UGT87A2, MFT, FRI, AGL57, TT16, and MAF4.

### 3.2. Plant Height-Related QTLs and Genes

Some Rht loci have been suggested to negatively affect anther extrusion [44,45]. The insertion of Rht genes was crucial for the success of the “green revolution” in wheat. Replacement of some widely used Rht loci with alternative variants more neutral for the flowering process may be recommended in hybrid breeding [46]. For example, the Rht1 and Rht24 genes reduce plant height, but Rht1 simultaneously reduces anther extrusion, whereas Rht24 does not have such adverse effects [30]. All wheats are assumed to be monomorphic for Rht-A1a [47]. Rht24 occurs at relatively high frequencies in European and Chinese wheat cultivars and was mapped to the same region as Rht14, Rht16, and Rht18 [48,49].

QTLs and candidate genes for PH were collected from the catalog of gene symbols for wheat ([47] with updates), and 44 microarray probes reported for PH [50] were mapped to separate physically linked regions in the Chinese Spring genome V 1.0 (Table S7A). We found eight DArTseq markers overlapping the four regions identified with these microarray probes. Additionally, DarTseq markers were found in the physical regions of QHt.nau-2D, Rht8, Rht13, and Rht22 [51,52,53].

Information on known genes responsible for wheat PH was used to search for homologous and paralogous genes (with identity to target > 50%). Sequences (TraesCS4A02G271000, TraesCS4B02G043100, and TraesCS4D02G040400) corresponding to gibberellic acid insensitive (GAI) Rht-A1, Rht-B1, and Rht-D1 were mapped to the 4AL, 4BS, and 4DS chromosome arms [54,55]. These GAI loci were highly conserved, and a search with blastn revealed no additional candidate homologous genes.

The remaining Rht genes were sensitive to GA. Two known genes from this group are GA2-β-dioxygenase (GA2oxA9) and AP2-D (Q gene), which correspond to Rht18 and Rht23 loci, respectively [50,56]. Two GA2oxA9 homologs and three paralogs were found, and five DArTseq markers coincided with the GA2oxA9 homolog located on the 6B chromosome. Two sequences were used to identify AP-2 homologs. Five AP-2 homologs were found, three of which were located in proximity (<5 Mbp) to 14 DArTseq markers associated with PH (Table S7A).

We identified 224 genes (containing 313 SNPs) assigned to DNA sequences related to PH using the text search in the description of genes by GO terms and Interpro features for the texts “gibberellin,” “auxin,” “cytochrome,” “kaurene,” “kaurenoic,” and “DELLA.” Most detected hits were for “cytochrome,” as in [50]. Of these, only 12 genes (with 14 SNPs) associated with PH showed noticeable negative or positive phenotypic effects. Three of them were orthologs of the A. thaliana genes CYP85A1, CYP85A2, CYP84A4, CYP84A1, and CYP734A1.

### 3.3. Spike Traits Related to QTLs and Genes

The positions of significant SNPs for grain weight, number per spike, and TKW were compared with those reported in previous studies (Table S7B). Maintaining a higher KN is an important breeding target for stress-tolerant lines [57]. A total of 142 regions were associated with KN, and 11 overlapped (±1 Mb) with the DArTseq markers reported in our study. Only precise QTLs spanning regions not exceeding 5 Mb were included in the search for common positions with SNP markers identified in our study. Ninety-two markers associated with kernel weight have been described in the literature, and only one marker had a position congruent with the SNP identified in our study. Out of the 123 SNPs or QTL described as responsible for TKW, five SNP markers were identified in previously described regions. TaGW8-B1 is associated with agronomic traits in bread wheat cultivars. The TaGW8-B1a allele increases TKW and spikelet number per spike and provides higher yields than cultivars with the TaGW8-B1b allele [58]. KASP markers for TKW have also been developed [59,60,61,62].

### 3.4. SNP Translation Effects

Among the identified polymorphisms, two SNPs had high translational effects. The first identified polymorphism, 1090593|F|0-44|CT, was associated with TKW and located in the gene TraesCS4B02G086500, annotated with the GO term “polygalacturonase activity”. Polygalacturonases are hydrolyzing enzymes implicated in a wide range of plant developmental processes, such as cell elongation, organ abscission, fruit ripening, microspore release, and pollen tube growth [63,64]. Members of two out of the five clades, C and F, are also expressed in grasses during root and seed development [65]. They were detected in the outer pericarp and intermediate layers of grains of the related species Aegilops tauschii [66]. Ye et al. [67] found three putative candidate genes for QTL mapped on chromosome 4A in wheat; two (TraesCS4A02G229600 and TraesCS4A02G229700) were orthologous to the Arabidopsis gene At2g43860, coding for polygalacturonase. SNP 1090593|F|0-44|CT was localized in the same homologous group but on chromosome 4B and had a high translational level, resulting in a STOP codon instead of a lysine codon. It is predicted that this substitution resulted in protein shortening (by 40 residues) and an altered C-terminus (the structures of wild-type and altered proteins, Figure S5).

The second SNP (1130302|F|0-44|TG) was located in gene TraesCS6A02G085100 (Arabidopsis RPT3 ortholog) with a high translational effect and annotated with the GO term “protein ubiquitination” and was associated with FT. Protein ubiquitination is a sophisticated system of post-translational modification in all eukaryotes and has been demonstrated to play a key role in various plant developmental stages and processes, such as seed dormancy and germination, root growth, flowering time control, self-incompatibility, chloroplast development, and several abiotic stress responses [68]. The SNP 1130302|F|0-44|TG was positioned in the intron and localized in the 5′UTR without any direct impact on the protein sequence. In general, cis-acting elements present in UTRs are essential for post-transcriptional control, including alternative polyadenylation, riboswitching, short-peptide translation, nonsense-mediated decay, and alternative splicing [69]. However, the T/G mutation found in our study may disrupt splicing, possibly resulting in the retention of this intron. Intron retention in the 5′UTR affects gene expression (by alternative splicing, alternative polyadenylation, and protein translation). Such an effect has been shown, inter alia, in Arabidopsis ZIF2 [70], EF1α-A3 [71] and rice OsmiR156h [72]. It has been postulated that UTR-related regulation of gene expression helps plants adapt to environmental fluctuations [69]. Flowering time is a major factor in climatic adaptation [73,74,75].

## 4. Materials and Methods

### 4.1. Germplasm Resources

The study was conducted using 509 wheat varieties and breeding lines. A set of 277 European varieties, registered mainly in Germany, Poland, and the United Kingdom, was used. Advanced breeding lines were represented by 232 accessions from the ongoing programs of the Plant Breeding Strzelce (STH) and Poznań Plant Breeding (PHR) companies. The genetic characteristics of the studied resources were provided by Tyrka et al. [28].

### 4.2. Field Phenotyping

Field experiments were conducted during two vegetation seasons, 2017/18 and 2018/19. Trials were established at four experimental stations of two breeding companies (STH and PHR) located in Strzelce (GPS: 52.3149° N, 19.4025° E), Kończewice (GPS: 53.1848° N, 18.5637° E), Nagradowice (GPS: 52.3178° N, 17.1529° E), and Leszno/Antoniny (GPS: 51.8586° N, 16.5902° E). Two replications of the studied objects were performed at each location. They were distributed within three randomized blocks. Each plot had an area of 1 m^2^. Cultivation conditions in brief: Sowing rate: 250 kernels per m^2^. Fertilization: 45 kg ha^−1^ of P_2_O_5_ and 80 kg ha^−1^ of K_2_O prior sawing followed by 150 kg of N per hectare applied in 2 doses during the vegetation season. Plant protection: only herbicides and insecticides were applied (no treatments against fungi and lodging). The following traits were analyzed in the field experiments:

Time of heading (GS55)—HT (number of days since May 1)

Time of flowering (GS65)—FT (number of days since May 1)

Plant height—PH (average height of plants on a plot in cm)

Number of kernels per spike—KN (assessed on 10 randomly collected spikes in each plot)

Weight of kernels per spike—KW (assessed on 10 randomly collected spikes in each plot; results in grams)

Thousand kernel weight—TKW (average from three random samples per plot, each containing 100 kernels)

To validate the results obtained from the HYBRE experiments, publicly available data from Polish state post-registration trials (www.coboru.gov.pl, accessed on 23 June 2019 [76]) from 2015–2018 were used. Only data on HT, PH, and TKW were available in the official trial reports among the six traits studied in our research.

### 4.3. Genotyping

A detailed description of genotyping and genotypic data processing was provided by Tyrka et al. [28]. Briefly, for each genotype, DNA was isolated from 15–20 bulked 2-week-old seedlings. DNA concentration and purity were determined using a NanoDrop spectrophotometer (Thermo Fisher Scientific, Waltham, MA, USA), and DNA quality was assessed using 1.5% agarose gel electrophoresis. The DNA was stored at −20 °C and diluted to a working concentration of 50 ng/μL for subsequent wheat DArTseq 1.0 genotyping completed by Diversity Arrays Technology (Bruce, Australia). Only selected high-quality data for 13,499 SNPs were taken into account in this research (dominant markers of the Silico-DArT type were omitted).

### 4.4. Data Analysis

Phenotypic data were analyzed using a linear mixed model (LMM) with fixed effects of the year (Y), location (L), Y × L interaction, and random effects of genotypes (G), G × Y interaction, and G × L interaction. Broad-sense heritability was estimated by the method described by Cullis et al. [77]. The additive main effects and multiplicative interaction (AMMI) analysis was performed as described by Gauch [78]. Genome-wide association study (GWAS), using SNP polymorphisms and genotypic means (BLUPs) obtained in individual experiments, was performed by a method that allows for the interaction of genetic effects with the environment, developed by van Eeuwijk et al. [79] and Malosetti et al. [35]. This is based on a linear mixed model with the population structure estimated by eigenanalysis of the kinship matrix (see [28]) and the compound symmetry variance-covariance model used for environmental variation. Marker data used for these analyses were coded as follows: 0, reference (REF) homozygote; 1, heterozygote; and 2, alternative (ALT) homozygote. Standard GWAS considers the additive marker effects, but also non-additive effects might explain an important proportion of the variation in complex traits ([80]). Therefore, GWAS for heterosis effects, estimated as the difference between the mean for heterozygotes and the mean for two homozygotes, was performed based on mean values over all experiments. To do this, marker data coded with 0, 1, and 2 values in the model matrix (used in GWAS for allelic substitution effects) were recoded to values of 0 for homozygotes (of both types) and 1 for heterozygotes [80]. The population structure was represented in the model by the eigenanalysis scores. The effects of marker linkage disequilibrium (LD) clusters were analyzed using mean trait values over all experiments and analysis of covariance, with the genotypic combinations in these clusters as classifying factors and eigenscores as covariates. *p*-values for allelic substitution effects and heterosis effects were corrected for multiple testing using the Benjamini–Hochberg (BH) method. Only SNP data with more than five accessions present in both homozygous classes were used during the genome-wide association analyses. An SNP effect was considered statistically significant if the BH-corrected *p*-value was lower than 0.05.

Statistical analyses were performed using Genstat for Windows 19th edition [81]. Visualizations of results were performed using Genstat 19 or R software. The annotation of SNP markers with respect to genomic positions, neighboring genes, their Gene Ontology classification, and SNP translation effects [29] used in this study were the same as those used by Tyrka et al. [28].

## 5. Conclusions

In conclusion, by employing an appropriate experimental and statistical approach, we generated a set of SNP markers that can be used in breeding practice to predict the earliness and height of plants to assess whether a given genotype will be particularly good as a maternal (early and short plant) or paternal form (late and tall plant). The estimated heritability of phenological traits and plant height was relatively high; therefore, selection based on the markers indicated during this study may be successful. In contrast, the heritability of yield-related traits in our field trials was relatively low; nevertheless, it remained within the range reported by other authors [82,83,84,85].

The main achievements of the presented work are:

1. The successful parallel selection of homozygous parental genotypes (based on traits regulated by additive genes controlling phenology and plant height) and components revealing a high heterosis effect (the choice based on the highest values of spike-yield-related traits revealed by heterozygous genotypes) using GWAS.
2. Demonstrating that the application of clustered markers for the choice of genotypes in multi-feature processes may be more efficient than classic selection based on single marker polymorphism (SNP)
3. Validation of the GWAS results using post-registration trial data.

## Figures and Tables

**Table 1:** Mean values, coefficients of variation (CV), variance components (with standard errors) for genotypes (G) and their interactions with year (Y) and localization (L), and broad-sense heritability coefficients for the six traits.

Trait	Mean	Minimum	Maximum	CV	Variance Component ± s.e.				Heritability (%)
					G	G × Y	G × L	Error	
HT	25.45	10.5	34.44	13.8	6.06 ± 0.42	0.91 ± 0.07	0.36 ± 0.03	1.56 ± 0.03	90.41
FT	31.67	17.87	39.95	11.74	4.04 ± 0.32	1.39 ± 0.11	0.46 ± 0.04	1.38 ± 0.03	80.77
PH	81.5	42.66	127.73	17.42	32.52 ± 2.37	5.92 ± 0.55	2.41 ± 0.31	22.15 ± 0.42	86.79
KN	62.24	27.69	103.9	15.88	14.05 ± 1.73	9.68 ± 1.12	11.44 ± 1.03	63.5 ± 1.2	54.60
KW	2.53	1.03	4.33	19.49	0.018 ± 0.003	0.028 ± 0.003	0.023 ± 0.002	0.14 ± 0.003	38.79
TKW	41.87	23.86	55.96	12.03	4.36 ± 0.56	5.41 ± 0.42	2.25 ± 0.18	9.72 ± 0.18	52.97

**Table 2:** Characteristics of allelic substitution effects for SNPs associated significantly with phenotypic traits.

Trait	Number of Associations	Negative Significant Effects		Positive Significant Effects		2th Percentile	
		Min	Max	Min	Max	Lower	Upper
HT	4206	−1.57	−0.0024	0.0003	1.49	−0.68	0.72
FT	4108	−1.30	−0.0027	0.0014	1.13	−0.61	0.64
PH	1999	−4.24	−0.0006	0.0013	3.18	−1.72	1.63
KN	1858	−4.29	−0.0015	0.0113	3.27	−1.36	1.54
KW	738	−0.09	−0.0001	0.0002	0.12	−0.05	0.05
TKW	1908	−1.41	−0.0002	0.0001	1.19	−0.66	0.61

**Table 3:** GE interaction and distribution of trait-associated SNPs in linkage blocks, subgenomes, and genes.

Trait	Number of Associations	Number of SNPs with Mean LD <0.01	Number of SNP Clusters	With GE Interaction (% out of Significant)	% in Subgenome			% in Genes	Number of SNPs Affecting Protein Translation			
					A	B	D		High	Low	Moderate	Modifier *
HT	444	71	220	66.0	47.1	36.9	13.3	55.4	0	57	45	342 (144)
FT	460	78	238	66.1	43.7	34.8	18.7	56.7	1	64	38	357 (158)
PH	391	21	123	38.1	20.2	63.2	13.3	59.6	0	52	66	273 (115)
KN	170	25	94	31.2	44.1	32.4	21.8	55.9	0	18	20	132 (57)
KW	51	10	36	68.6	41.2	39.2	19.6	54.9	0	7	6	38 (15)
TKW	272	22	115	61.4	55.1	36.4	5.9	58.5	1	23	29	219 (106)

* Total number of markers indicated by VEP [29] (in brackets: number of markers assigned to specific genes).

**Table 4:** Markers with concordant effects for sets 1 and 2 (in bold) and assigned to genes. Types of translation effects caused by substitution are provided (LOW, MDR—moderate, or MFI—modifier).

Marker	Gene	Effect	Interpro Description
**2253029|F|0-10|CT (negative)**	TraesCS2A02G482200	MFI	NAD-dependent epimerase/dehydratase; NAD(P)-binding domain superfamily
3938110|F|0-10|CG	TraesCS2B02G045100	LOW	NB-ARC;P-loop containing nucleoside triphosphate hydrolase; Leucine-rich repeat domain superfamily
**1237800|F|0-13|CG (positive)**	TraesCS2B02G164700	MFI	F-box-like domain superfamily
4910338|F|0-15|GC	TraesCS2B02G475700	MDR	Zf-FLZ domain; Zf-FLZ domain
2322929|F|0-10|AT	TraesCS2B02G490600	MFI	Ribonuclease H-like superfamily; Exonuclease, RNase T/DNA polymerase III; Ribonuclease H superfamily
2322355|F|0-40|CG	TraesCS2B02G521100	LOW	Glycosyl transferase;1,3-beta-glucan synthase subunit FKS1-like, domain-1
1238701|F|0-16|GA	TraesCS2D02G127300	LOW	F-box domain;F-box-like domain superfamily
1675478|F|0-15|TG	TraesCS3A02G506600	LOW	NAD(P)-binding domain superfamily; NAD(P)-binding domain superfamily
7350269|F|0-8|TC	TraesCS3A02G517700	LOW	
1049114|F|0-20|AG	TraesCS3D02G511900	MFI	Ubiquitin-like domain; Ubiquitin domain; Ubiquitin-like domain superfamily
1675534|F|0-35|AC	TraesCS3D02G513900	MDR	UDP-glucuronosyl/UDP-glucosyltransferase
2252787|F|0-19|CT	TraesCS3D02G529700	MFI	Coenzyme Q-binding protein COQ10, START domain; START-like domain superfamily
7352843|F|0-43|AT	TraesCS3D02G531000	MDR	Transcription initiation factor IIA, gamma subunit; Transcription factor IIA, helical; Transcription factor IIA, beta-barrel; Transcription initiation factor IIA, gamma subunit, C-terminal
7352096|F|0-35|GC	TraesCS3D02G541900	LOW	Uncharacterised conserved protein UCP015417, vWA
7353078|F|0-15|CA	TraesCS3D02G542800	MDR	Oxoglutarate/iron-dependent dioxygenase; Non-haem dioxygenase N-terminal domain; Isopenicillin N synthase-like
3024403|F|0-23|CT	TraesCS3D02G543100	LOW	DnaJ domain; Tetratricopeptide-like helical domain superfamily; Tetratricopeptide repeat-containing domain
1009915|F|0-65|CG	TraesCSU02G059500	LOW	Leucine-rich repeat, cysteine-containing subtype; SKP1/BTB/POZ domain superfamily; BTB/Kelch-associated; Leucine-rich repeat domain superfamily
1056528|F|0-9|TG (negative) *	-	MFI	-

* marker not in the gene but showing congruent association with HT, FT, and PH.

**Table 5:** Characteristics of heterosis effects for the five traits (KW was omitted due to the lack of significant effects).

Trait	Number of Significant Effects	% in Genome			% in Genes	Number SNPs with Protein Translation Effect (in Genes)		
		A	B	D		Low	Moderate	Modifier *
HT	437	33.9	28.6	35.5	57.7	106	52	275 (94)
FT	393	35.4	28.0	35.4	57.0	96	45	249 (83)
PH	324	35.5	31.8	28.7	52.8	52	41	229 (78)
KN	62	37.1	32.3	29.0	62.9	18	3	41 (18)
TKW	45	42.2	20.0	37.8	68.9	13	2	30 (16)

* Total number of markers indicated by VEP [29] (in brackets: number of markers assigned to specific genes).

## Data Availability

The data presented in this study are available in Supplementary Materials.

## Supplementary Materials

The following supporting information can be downloaded at: https://www.mdpi.com/article/10.3390/ijms232315321/s1.

## Abbreviations

AMMI	Additive main-effects and multiplicative interaction
BLUP	Best linear unbiased prediction
FT	Time of flowering
GBS	Genotyping by Sequencing
GS	Growth stage (BBCH scale)
GWAS	Genome-wide association studies
HYBRE	The project acronym and the name of the consortium
HT	Time of heading
KN	Number of kernels per spike
KW	Weight of kernels per spike
PH	Plant height
PRT	Post-registration trials
SNP	Single Nucleotide Polymorphism
TKW	Thousand kernel weight
VEP	Variant Effect Predictor
