# Supplementary material for: Genome-Wide Association Analysis for Hybrid Breeding in Wheat
Source: Int J Mol Sci. 2022 Dec 5;23(23):15321. doi: 10.3390/ijms232315321 (PMC9740285; doi:10.3390/ijms232315321)
Supplement: Supplementary file 1 [file ijms-23-15321-s001.zip › supplementaryFiles/SupplementaryFigures.pptx]

## Slide 1
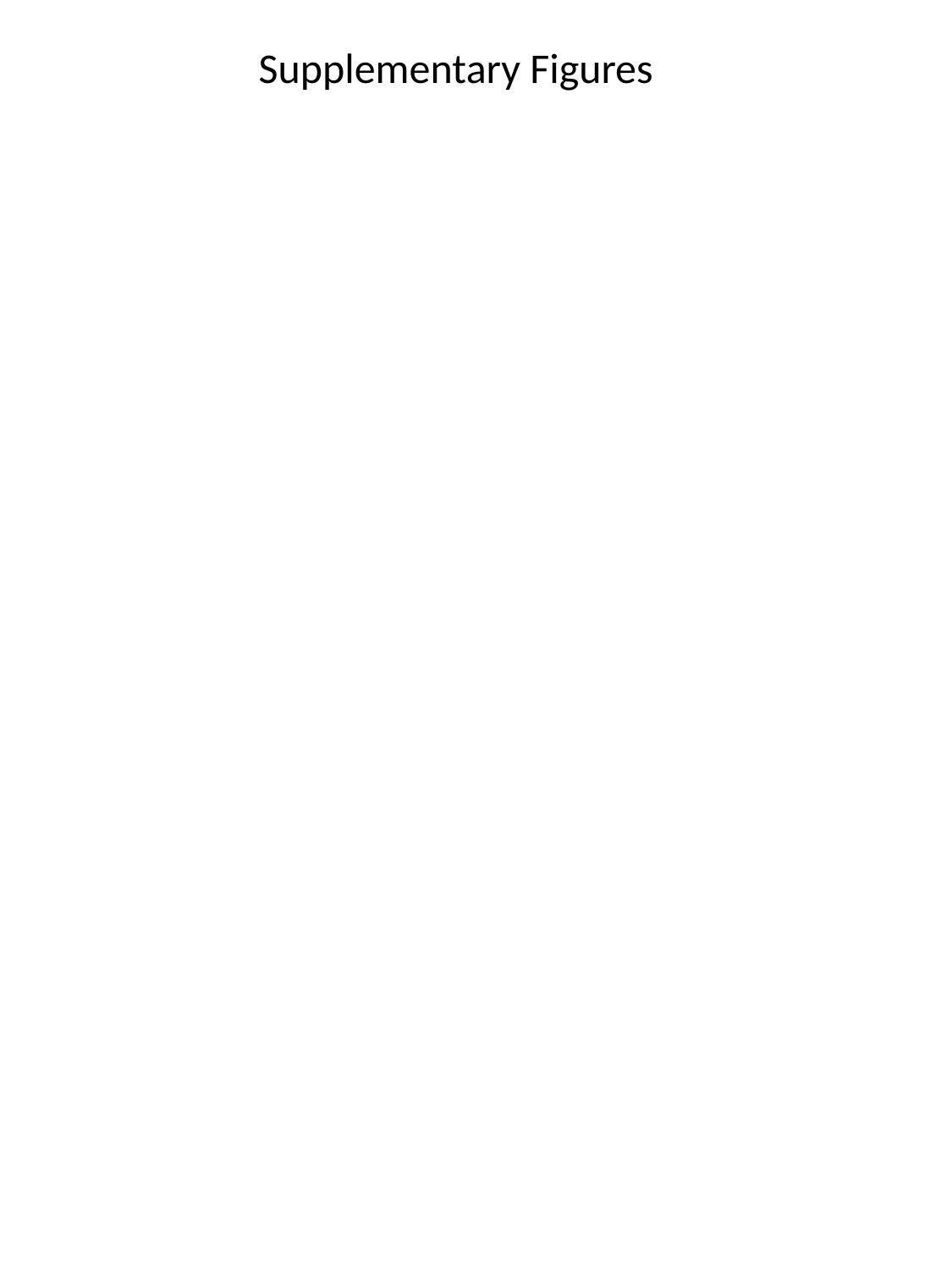

Supplementary Figures

## Slide 2
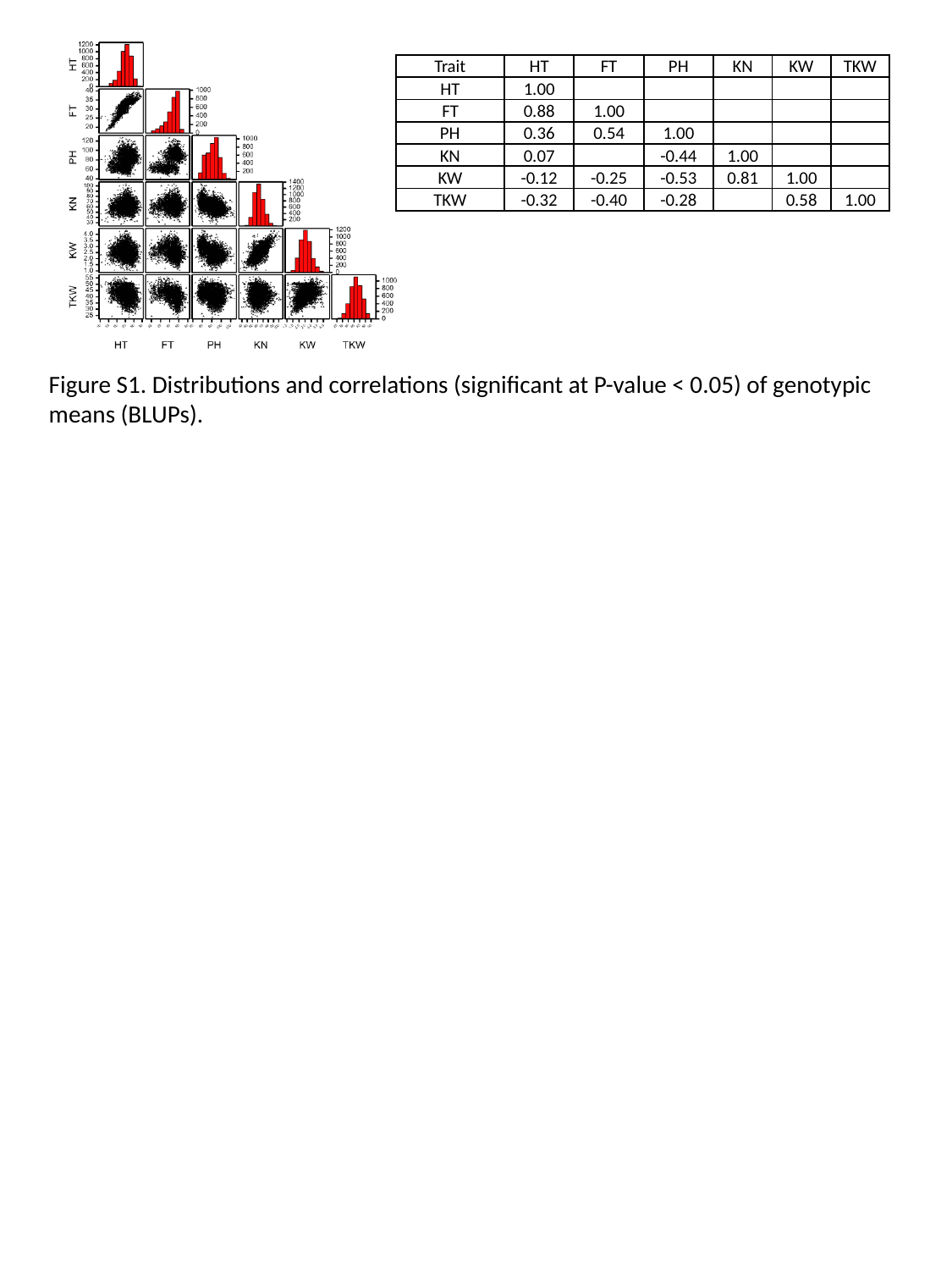

| Trait | HT | FT | PH | KN | KW | TKW |
| --- | --- | --- | --- | --- | --- | --- |
| HT | 1.00 | | | | | |
| FT | 0.88 | 1.00 | | | | |
| PH | 0.36 | 0.54 | 1.00 | | | |
| KN | 0.07 | | -0.44 | 1.00 | | |
| KW | -0.12 | -0.25 | -0.53 | 0.81 | 1.00 | |
| TKW | -0.32 | -0.40 | -0.28 | | 0.58 | 1.00 |
# Figure S1. Distributions and correlations (significant at P-value < 0.05) of genotypic means (BLUPs).

## Slide 3
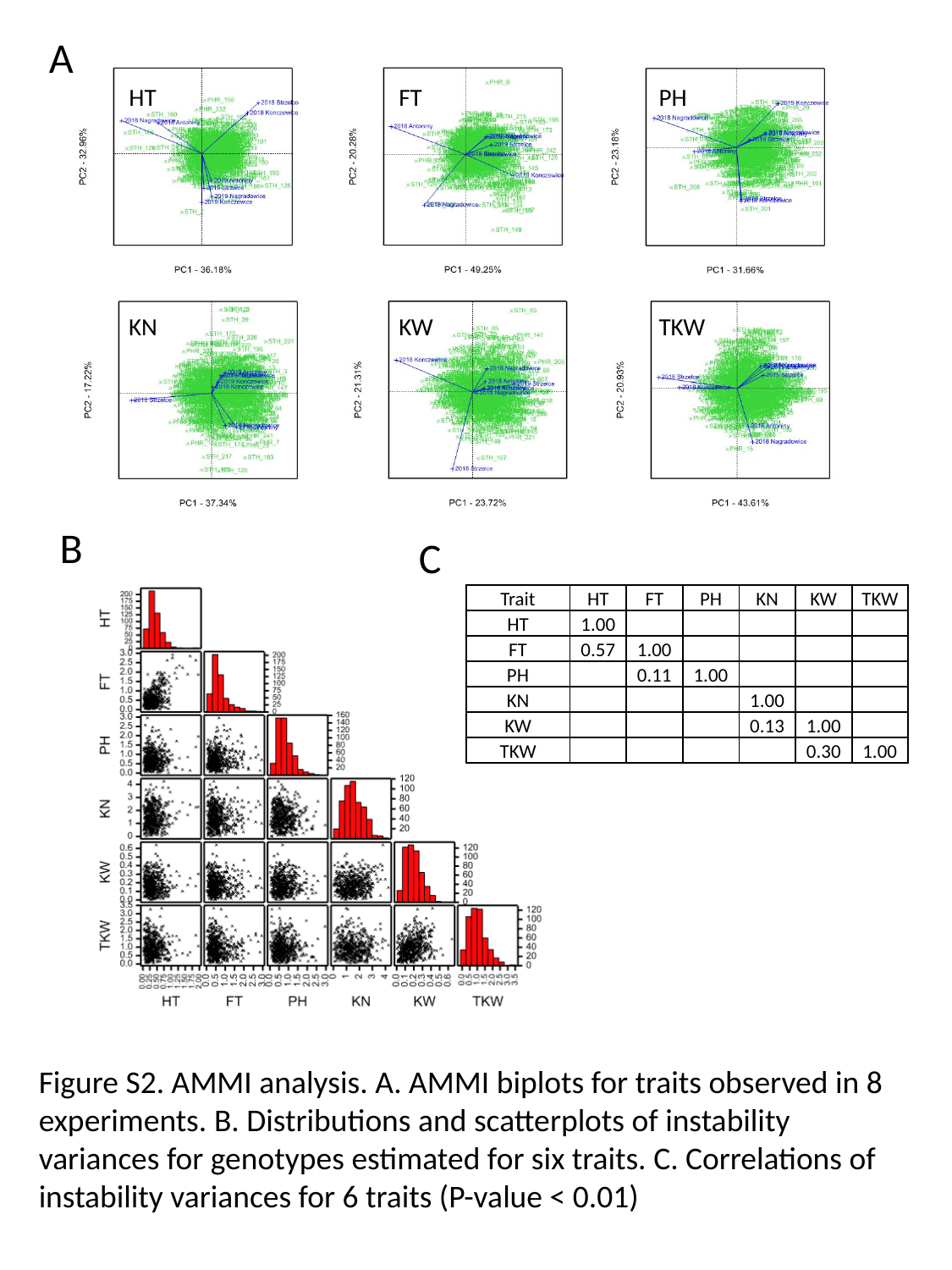

A
HT
FT
PH
KN
KW
TKW
B
C
| Trait | HT | FT | PH | KN | KW | TKW |
| --- | --- | --- | --- | --- | --- | --- |
| HT | 1.00 | | | | | |
| FT | 0.57 | 1.00 | | | | |
| PH | | 0.11 | 1.00 | | | |
| KN | | | | 1.00 | | |
| KW | | | | 0.13 | 1.00 | |
| TKW | | | | | 0.30 | 1.00 |
Figure S2. AMMI analysis. A. AMMI biplots for traits observed in 8 experiments. B. Distributions and scatterplots of instability variances for genotypes estimated for six traits. C. Correlations of instability variances for 6 traits (P-value < 0.01)

## Slide 4
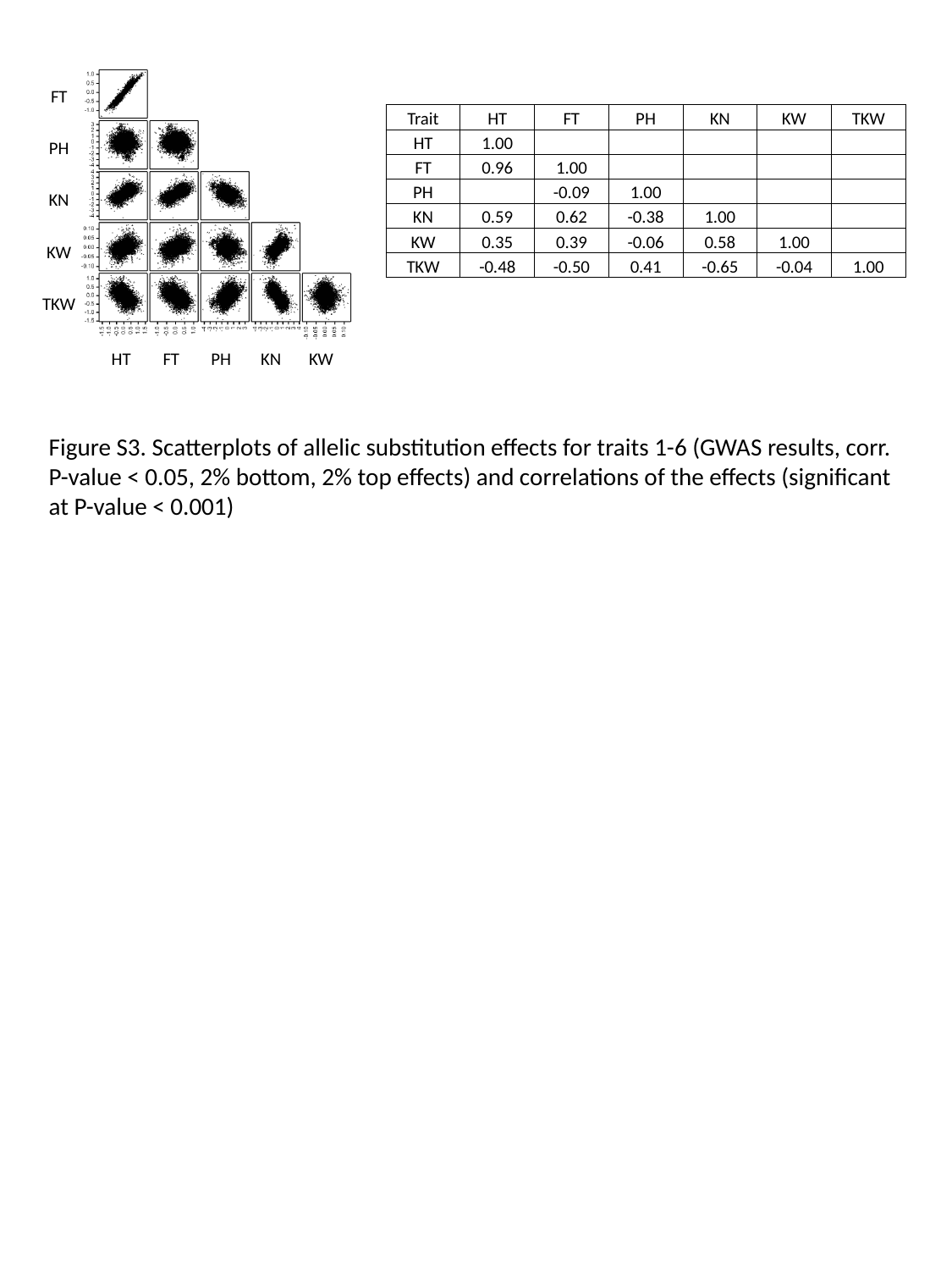

| FT |
| --- |
| PH |
| KN |
| KW |
| TKW |
| Trait | HT | FT | PH | KN | KW | TKW |
| --- | --- | --- | --- | --- | --- | --- |
| HT | 1.00 | | | | | |
| FT | 0.96 | 1.00 | | | | |
| PH | | -0.09 | 1.00 | | | |
| KN | 0.59 | 0.62 | -0.38 | 1.00 | | |
| KW | 0.35 | 0.39 | -0.06 | 0.58 | 1.00 | |
| TKW | -0.48 | -0.50 | 0.41 | -0.65 | -0.04 | 1.00 |
| HT | FT | PH | KN | KW |
| --- | --- | --- | --- | --- |
Figure S3. Scatterplots of allelic substitution effects for traits 1-6 (GWAS results, corr. P-value < 0.05, 2% bottom, 2% top effects) and correlations of the effects (significant at P-value < 0.001)

## Slide 5
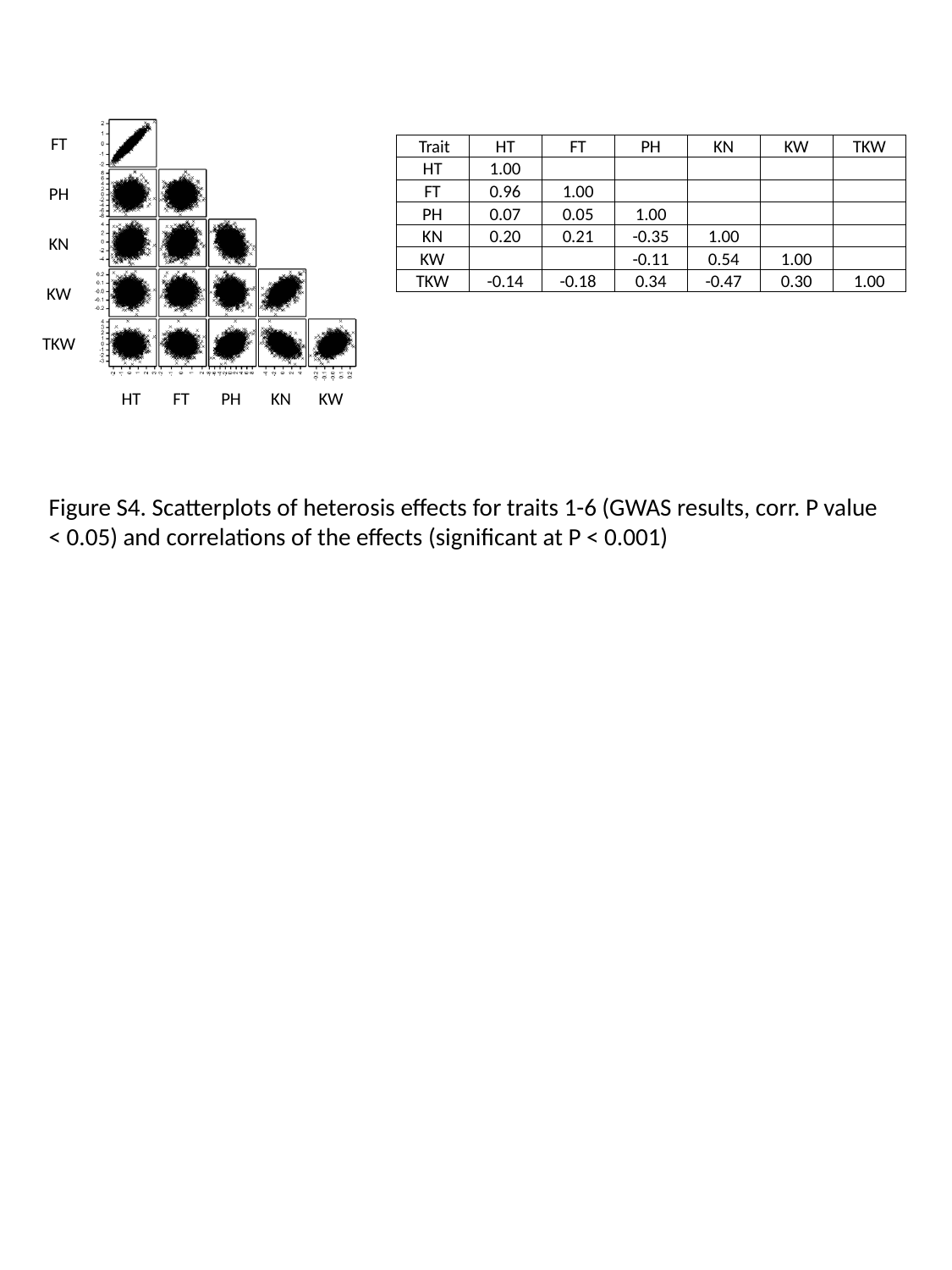

| FT |
| --- |
| PH |
| KN |
| KW |
| TKW |
| Trait | HT | FT | PH | KN | KW | TKW |
| --- | --- | --- | --- | --- | --- | --- |
| HT | 1.00 | | | | | |
| FT | 0.96 | 1.00 | | | | |
| PH | 0.07 | 0.05 | 1.00 | | | |
| KN | 0.20 | 0.21 | -0.35 | 1.00 | | |
| KW | | | -0.11 | 0.54 | 1.00 | |
| TKW | -0.14 | -0.18 | 0.34 | -0.47 | 0.30 | 1.00 |
| HT | FT | PH | KN | KW |
| --- | --- | --- | --- | --- |
Figure S4. Scatterplots of heterosis effects for traits 1-6 (GWAS results, corr. P value < 0.05) and correlations of the effects (significant at P < 0.001)

## Slide 6
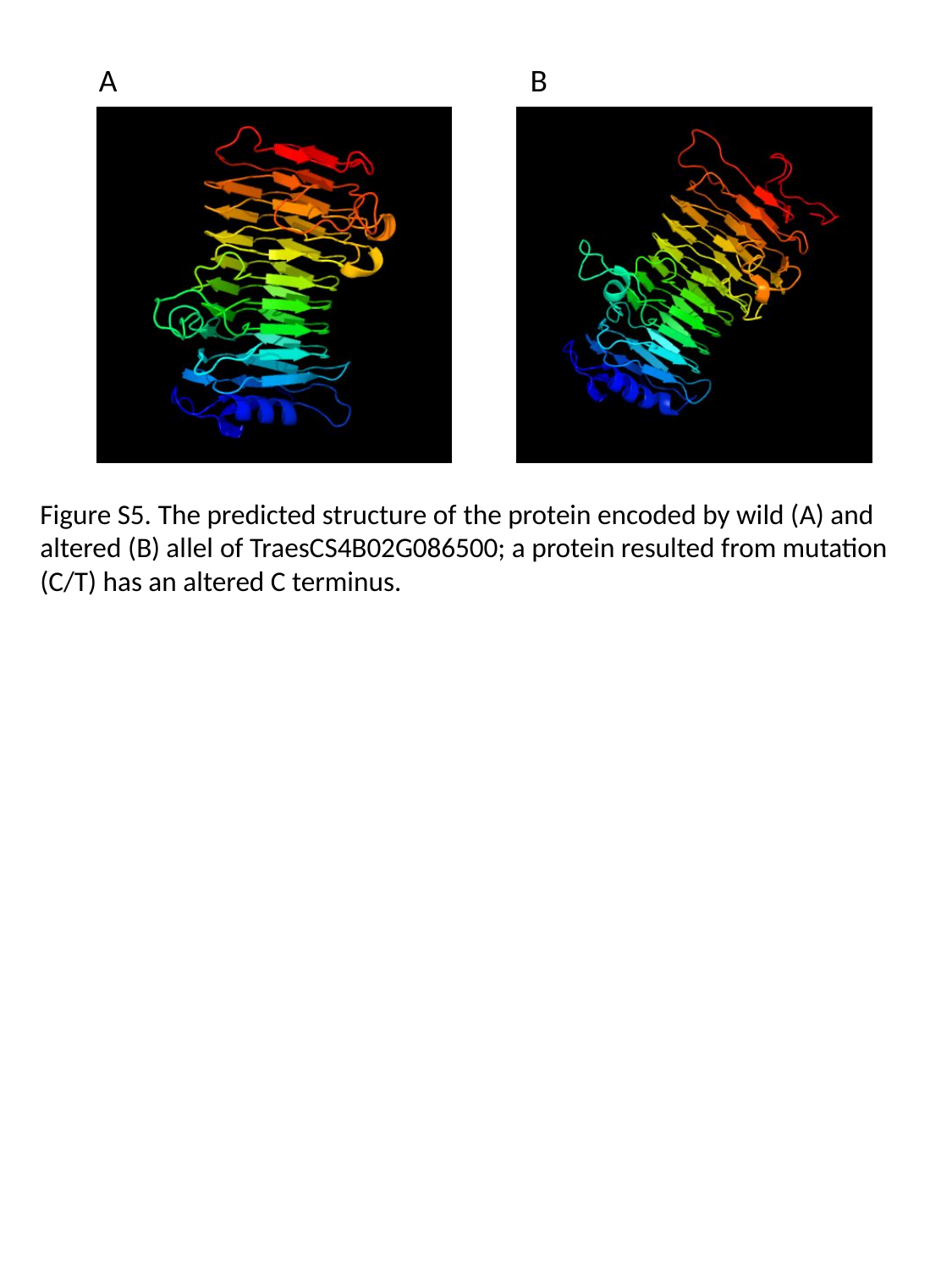

A
B
# Figure S5. The predicted structure of the protein encoded by wild (A) and altered (B) allel of TraesCS4B02G086500; a protein resulted from mutation (C/T) has an altered C terminus.
